# The Institutional Worker

**Published:** 1913-05-17

**Authors:** 


THE
### INSTITUTIONAL WORKER
**Being a Special Supplement to "The Hospital"**

### OUR BUREAU OF INFORMATION.

### RULES FOR CORRESPONDENTS.

1. Every letter, on whatever subject, must be accompanied by the coupon to be cut from the back cover (inside page) of THE HOSPITAL, current issue, and must contain the name and address of the correspondent with pseudonym for publication if desired.

2. Replies by post cannot be given, save under exceptional circumstances at the Editor's discretion.

3. Letters from Approved Homes in reply to special needs published in the Bureau should state terms and full particulars, and be sent prepaid under cover to the Editor of the Bureau with name written across coupon for identification.

4. Proprietors of Homes which have not yet been entered on the List of Approved Homes, but have spare accommodation likely to suit special needs, are invited to write for an application form for registration. The fee for registration, which includes two announcements of the Home in the Bureau and other privileges, is 10s.

5. All communications to be addressed to the Editor of THE HOSPITAL, 28 Southampton Street, Strand, London, W.C., and marked "Bureau of Information."

### INVITATION TO READERS.

**The Editor is prepared to make known without charge the needs of any who may be sick or in difficulties, and to guide them in making choice of Homes for treatment or convalescence. Reduced terms can often be arranged with Homes on the Approved List for those unable to pay full fees. Queries are specially invited from those who are engaged in any kind of philanthropical work. A new edition of the leaflet describing the purposes of the Bureau of Information is now ready and will be sent free of charge on application.**

### Convalescence from Infectious Disease.

THERE are certain restrictions at most convalescent institutions as regards the admission of convalescents from infectious diseases, but generally the cases are precluded only so long as they are liable to convey infection to others. At the Broadstairs (Children's) Branch of the Metropolitan Convalescent Institution, patients are received four weeks after convalescence. Patients are admitted upon the recommendation of a governor or subscriber. Application should be made to the Secretary, 14 Victoria Street, S.W.—"A. E. R."

### The Protection of Women and Children.

THE Associated Societies for the Protection of Women and Children may afford you some assistance. The main object of this Society is to protect women and children in cases of neglect, cruelty, outrage, desertion, and other wrongs by affording them advice or legal assistance, and, when deemed desirable, by prosecuting the offenders. It works, by special arrangement, in conjunction with the National Society for the Prevention of Cruelty to Children. Application should be made to the Secretary, 60 Haymarket, S.W. —"OPPRESSED."

### Co-operation as a Remedy.

THE treatment of children suffering from tuberculous joints and other conditions requiring a period of treatment more or less indefinite presents a difficulty at many of our general hospitals. We know that in many instances lack of accommodation and special provisions makes it impossible to treat these cases to a conclusion. Applications for the admission of such cases are numerous; and in the absence of more suitable provision certain of them are admitted for such periods as may be necessary for the relief of acute conditions; the cases are then discharged with special instructions or appliances. Whilst treatment such as this often does serve a useful purpose, yet it is, on the other hand, as often fruitless owing to the absence of adequate after-care. The method of dealing with such cases is, to say the least of it, haphazard, and points to a need for co-operation between the hospitals and other agencies, or to the establishment of such agencies as may be necessary for remedying the evil. The solution of this question should appeal to the economist as much as to the humanitarian, and we should be interested to learn the experiences of our readers and to have their suggestions.

### The Children's Aid Association.

THE work which is being carried on by the Invalid Children's Aid Association is an exceptional example of the power of personal service and co-operation. This Association, which assists the invalid and crippled children of the poor, works chiefly through visitors, each of whom takes charge of one or more children; for the primary aim of the Association is to find for every child referred to it, a friend who shall give to it, in so far as circumstances permit, liberal personal service. Skilled nursing, special medical advice, medical comforts and clothing, surgical appliances, and hospital and convalescent treatment are amongst the material benefits dispensed. Treatment continues generally up to the time the children attain the age of fifteen years, but this rule does not apply in the case of children learning trades, for one of the aims of the Association is to obtain for them the means of earning their livelihood. The Society has many branches around London, and the fact that it appreciates the usefulness of co-operation is indicated by its excellent federation scheme which now embraces many similar societies in the provinces which are represented on the Central Council by delegates. The exchange of special knowledge and experience, which is thus made possible, is of the greatest help in dealing with this difficult work, and in promoting its development. Many children requiring special help are annually brought to the notice of the Society through the hospitals, but there is still plenty of room for closer co-operation. Those interested may obtain a report of the Society upon application to the Secretary, 69-71 Denison House, 296 Vauxhall Bridge Road, Westminster, S.W.

### Treatment of Spinal and Joint Diseases.

WE would suggest that you should apply to the Secretary of the Birmingham Cripples' Union. This Society supports an excellent in-

### INSTITUTION FACTS AND FIGURES.

#### Question for May.

**During a period of four weeks in the winter months state what was the quantity of gas consumed in your institution for purposes other than illuminating, its approximate cost, the quantity of each kind of fuel used and its cost, deducting the cost of fuel for laundry purposes (if any).**

**In answering this question the following particulars should be given: (1) Number of wards and size of each (in beds); (2) total number of coal fires (*a*) in the wards, (*b*) in the rest of the building; (3) the number of gas-cooking and other stoves and sterilisers; (4) the name of the heating and hot-water supply service.**

**In the ensuing month contributors will be asked to give fuller particulars upon this subject by describing the character of the buildings, apparatus, etc.**

NOTE.—The figures must be the result of actual observation and computation, not estimates. The The best answers will be published, and for each contribution considered by the Editor to be of sufficient interest for publication payment of FIVE SHILLINGS will be made.

RULES.

The following rules must be observed by all those answering the Facts and Figures Questions:—

1. Answers must be written on one side of the paper. They must bear tne name and address of the sender and be accompanied by coupon to be cut from the back cover (inside page) of the current issue of THE HOSPITAL. A pseudonym must be chosen if the name is not to be published.

2. Answers must be addressed to the Editor of THE HOSPITAL, 28-29 Southampton Street, Strand, London, W.C.; they must be marked in the left-hand corner "Facts and Figures," and must be received before the end of the month.

### Trained Masseuse.

WE presume that you hold the Certificate of the Incorporated Society of Trained Masseuses. This Society has a registry for members, and it is possible that it may be able to help you in the direction you wish. Apply to the Secretary, 12 Buckingham Street, Strand, W.C. You should also study the advertisements of the nursing journals.—"J. P."

### Maternity Training.

Apply to the London County Council for particulars of scholarships, which are offered periodically for midwifery training. The terms for midwifery training at the District Nurses' Home, Howard's Road, Plaistow, are comparatively moderate. You will find a list of Dublin nursing institutions and hospitals in the book "How to Become a Nurse," published by the Scientific Press, Ltd., 28 and 29 Southampton Street, Strand, W.C. You should also study the advertisements in the nursing journals.—"Anxious, Dublin."

### Proprietary Remedy.

We are unable to advise you to place your ointment on the market. It is as contrary to the ethics of the nursing profession as of the medical profession to promote the sale of proprietary remedies. The reason is, doubtless, obvious to you; no remedy, however good, can be effective if used indiscriminately, as yours would be under the conditions suggested.—"V. D. P."

### Ward Economy.

Your complaint is a common one, but the difficulty can be quite easily overcome. A weekly return of antiseptics and drugs required for the ward should be made by the sister, and before these are supplied from the dispensary the items should be checked by an independent officer acquainted with the needs of the ward, and compared with the corresponding ones of previous weeks, due regard being paid to the numbers under treatment and the nature of the cases. It is especially desirable that the Sister of each ward should be supplied with a schedule giving the prices of the various drugs in use, for it not infrequently occurs that ignorance upon this subject leads to extravagance which would otherwise be guarded against.—"J. W."

### Absorbent Cotton.

The length of the fibre usually determines the price of absorbent cotton. A cheap absorbent cotton has a short fibre, or it may be that it is not thoroughly freed of oil. For surgical purposes a short-fibre cotton answers the purpose, although, in pads, it tends to "lump." The presence of oil may be tested by dropping a piece of the cotton into water. If oil free, it will fill and sink immediately. An oily absorbent cotton is dear at any price.—"Sister C."

stitution on the outskirts of Birmingham, where children suffering from diseases of the spine and joints are treated until cured, or until such time as they shall have obtained all the relief that is humanly possible. We understand that the Society's funds are insufficient to maintain all their beds, and it is possible, therefore, that they might consider a proposal to reserve a bed or beds for your cases for a certain annual subscription. "W. D. H."

### Disabled Nurse.

Apply to the Secretary, Trained Nurses' Annuity Fund, 73 Cheapside, E.C. Annuities of from 7s. to 10s. a week are granted by the Council to suitable candidates, as funds permit. The chief conditions and qualifications are that the candidate holds a certificate of full training in some recognised training school; that she has attained the age of forty years, except in extraordinary circumstances; and that she is incapacitated by ill-health or other sufficient cause from following her profession. There are a large number of waiting applicants, so that the prospect of immediate relief is small.—"B. M."

### Working Boys' Camps.

The season of the seaside camps for working boys living or working within the Diocese of London opens on July 12 next. The payment required varies in accordance with the age and wages of the boys—from 7s. 6d. to 15s. a week. Application should be made to the Secretary, London Diocesan Council for the Welfare of Lads, 22 Northumberland Avenue, W.C. The camp was founded in order to give a seaside holiday to hard-working London boys of good character, whose means are too small to allow of a real holiday without help.—"Minister."

### The Training of Midwives.

Apply to the Association for promoting the Training and Supply of Midwives, Dean Farrar Street, Westminster, S.W. This Society was formed to promote the training and supply of midwives to meet the requirements of the Midwives Act of 1902. It raises funds to facilitate the training of midwives in approved hospitals or other institutions. It also co-operates with other organisations which are working for the same object, and in certain instances makes grants towards the training of women as midwives.—"Nurse W."

### Special Baths for Treatment of Burns.

In the larger hospitals provision is sometimes made whereby severe cases of burns may be treated in water-baths. These baths must be fitted in such a way as will permit of a constant flow of water sustained at a temperature of 99° to 100° F. The patient is suspended in the water in a special form of hammock, and air or water cushions may be used to secure the patient's comfort. Though the treatment is expensive, for the reason that it entails constant supervision, it is undoubtedly justified even in a small hospital such as yours, situate as it is in a district where cases of severe burns are frequently occurring. In the treatment of children by this method it is of course comparatively easy to maintain a small portable bath at a fairly even temperature by hand; but it is quite impossible to deal with an adult in this way, and consequently the absence of a special bath precludes this form of treatment. An article on the use of the permanent bath appeared in The Hospital of April 19 last. It would be interesting to hear the experiences of others upon this subject.—"Progress."

### Metal Filament Lamps.

You will reduce breakage considerably if you will instruct the maids, when dusting the lamps, first to turn on the current. The metal filament does not break so readily when hot, as it is much more elastic.—"Housekeeper."

### Electrical Apparatus.

The repair of electrical apparatus presents a difficulty at most of the smaller institutions where the amount of work to be done does not justify the employment of an artisan with the necessary knowledge. You will not find that the makers of the apparatus are anxious to undertake small items of repair; and, moreover, reference to the makers on every occasion would involve considerable expense and inconvenience owing to the distance which separates you. We would suggest that, if you have a man on the premises with a slight mechanical and electrical knowledge, you should arrange with the makers to give him such instruction as will enable him to properly care for the apparatus; otherwise, the only alternative is to enter into a contract with a local firm of electricians, whereby they will make themselves acquainted with the apparatus, periodically examine it, and keep it in order.—"Secretary."

### Fittings and Furniture.

We would advise you to advertise your requirement for second-hand fittings and furniture for your Home in the Sales and Exchange columns of The Hospital.—"K. P."

### A Vacant Homestead for Convalescent Cases.

As regards your ideal house with two large covered-in verandahs and attractive grounds at Chine Side, Shanklin, which you inform us would serve as an excellent convalescent home, we would suggest that your best way of bringing it before the notice of the readers of The Hospital with a view to selling or letting would be to insert an announcement in the advertisement columns.—"A. M. M."

### The Editor's Letter-Box.

The Editor is indebted for testimonial received in response to inquiry from Miss Rickett-Smith.

M. Ward.—We are endeavouring to obtain for you the information you require.

A communication has been received and attended to from L. Whiteside.

Communications have been received from E. A. S. and Sister Hewitt, and are being attended to.

### Editor's Notices.

**Contributions:**

Contributions should be written, or preferably typed, on one side of the paper only, and all articles sent in are accepted upon the distinct understanding that they are forwarded to The Hospital only.

The Editor cannot undertake to return MSS. not used, but when a stamped directed envelope is enclosed the MSS. may be returned if a special request is made.

Accepted articles and paragraphs of news will be paid for after publication at the scale rate.

**Address:**

To prevent delay all contributions and letters on editorial business must be addressed exclusively to the Editor, "The Hospital," 29 Southampton Street, Strand, London, W.C. It is important that this regulation shall be strictly observed.

**Correspondence:**

Correspondence on all subjects is invited. The name and address of correspondents must be given as a guarantee of good faith, but not necessarily for publication.

**Special Articles:**

Special articles are invited, and questions, inquiries, and paragraphs upon all matters relating to the work, administration, and management of general, special, mental, fever, cottage and convalescent hospitals, sanatoria, homes, institutions, societies and organisations for the treatment or care of the sick, injured and dependants of all classes. Every matter affecting the interests and welfare of the staff of all grades working in these institutions will receive special consideration.

**Administrative Medicine, Research and Items of News:**

Special terms will be paid for approved articles on subjects in this wide field by experts who have made some section of it their special study and interest. We wish to give prominence to every movement tending to advance accuracy of diagnosis, efficiency in treatment, and the development of modern methods to eradicate disease and promote the welfare of the suffering.

**A Bureau of Information:**

We invite inquiries and applications for information and help in providing for that numerous class of sufferers who require special care or house-room which they cannot obtain in their own homes or secure for themselves. We cannot, however, prescribe, or recommend practitioners.

**Books for Review:**

Publishers are particularly requested to send advance proofs of any new books of importance whenever possible, as the Editor has made arrangements to publish immediate reviews on a new plan.

**Photographs, Plans, Blocks, and Illustrations:**

It is requested that wherever possible MSS. may be accompanied by illustrations in any of the above forms. The name of the sender and of the article to which it belongs should be written on the back of each photograph, drawing, or block for purposes of identification.

**Local Papers:**

Newspapers containing reports or news paragraphs should be marked and addressed to the Sub-Editor.

**The Coupon System:**

A coupon will be found at the bottom of the third inside page of the cover each week. This coupon must be attached to every question or inquiry to which an answer is desired in The Hospital.

### To Fill that Vacancy.

As a medium for announcing vacancies in every department of Institutional Life, the exceptional value of *The Institutional Worker* is evidenced by the large number of such notices that appear week by week in this section of The Hospital. No more adequate proof of the usefulness of *The Institutional Worker* can be desired than the fact that the Responsible Officers of the most important Institutions in the United Kingdom employ its columns again and again whenever they have a vacancy to fill upon their staffs. It also provides exceptional facilities for every class of advertisement affecting Institutional Life and Work, and as a means of securing re-engagements in any section of the Institutional, Health, and Sanitary Services it is absolutely incomparable, since no other journal possesses such a wide and comprehensive circulation in these directions as The Hospital. If you are looking for a post an announcement of twenty-one words, costing the small sum of 1s., may save you many hours of anxious thought, and will certainly bring your requirement immediately before those most likely to need your services.

This form may be used for Appointments Wanted advertisements, and when filled up should be posted to

*The Manager*, The Hospital,
28 and 29 Southampton Street,
Strand, W.C.

Appointments Wanted, 21 words ... 1s. 0d.

| | | |
|---|---|---|
| | | |
| | | |
| | | |
| | | |
| | | |
| | | |
| | | |

### Manager's Notices.

**Letters relating to the publication, sale, and advertising departments of "The Hospital" must be addressed to The Manager, "The Hospital," The Hospital Building, 28 and 29 Southampton Street, London, W.C.**

**Subscriptions** may begin at any time, and are payable in advance. Cheques and Post-Office Orders should be crossed London County and Westminster Bank, Covent Garden Branch, and made payable to the Manager of The Hospital.

Rates of Subscription (including Postage).

| *United Kingdom.* | | *Foreign and Colonial.* | |
|---|---|---|---|
| Three Months ... ... | 2s. 0d. | Three Months ... ... | 2s. 6d. |
| Six Months ... ... | 4s. 0d | Six Months ... ... | 5s. 0d. |
| Twelve Months ... ... | 6s. 6d | Twelve Months ... ... | 8s. 8d. |

**Advertisements:**

To ensure insertion, all advertisements for The Hospital must reach the Manager not later than Wednesday morning in each week. For scale of charges see pages iii and 4.

**Remittances:**

**It is especially requested that remittances be made by Cheque or Postal Order, and in halfpenny stamps only when the amount is under sixpence.**

LATEST EDITION

OF

THE NURSE'S PRONOUNCING DICTIONARY

OF

MEDICAL TERMS AND NURSING TREATMENT

Compiled by

HONNOR MORTEN

Seventh Edition.

Revised and Edited by

Mary I. Burdett.

BECAUSE:

IT IS QUITE UP TO DATE.

CONTAINS NO OBSOLETE TERMS.

IS ABSOLUTELY DEPENDABLE.

The proofs were passed by a highly Qualified Medical Man, consequently the utmost reliance can be placed in the information within its pages.

GET A COPY TO-DAY.

Of all Booksellers, or direct from THE PUBLISHERS,

THE SCIENTIFIC PRESS, Ltd.,
28 & 29 Southampton Street,
Strand, London, W.C.

